# Comparison of pemetrexed plus cisplatin with gemcitabine plus docetaxel in refractory/metastatic osteosarcoma: Clinical outcomes from a retrospective database monitored in a single institute

**DOI:** 10.3892/ol.2014.2472

**Published:** 2014-08-21

**Authors:** WEN-XI YU, LI-NA TANG, FENG LIN, YANG YAO, ZAN SHEN

**Affiliations:** Department of Oncology, Affiliated Sixth People’s Hospital, Shanghai Jiaotong University, Shanghai 200233, P.R. China

**Keywords:** osteosarcoma, metastatic, gemcitabine, docetaxel, pemetrexed, cisplatin

## Abstract

The prognosis for patients with relapsed/metastatic osteosarcoma is poor and the optimal treatment strategy remains to be refined. Whilst gemcitabine plus docetaxel combination treatment has already been demonstrated to have certain promising results in the treatment of osteosarcoma, the use of pemetrexed, a multi-targeted antifolate, remains controversial. In the present study, a retrospective investigation was conducted to evaluate the toxicity and efficacy of the pemetrexed plus cisplatin combination in relapsed/metastatic osteosarcoma. Comparison of this treatment with that of the gemcitabine plus docetaxel combination was also conducted. Clinical data from 39 patients suffering from refractory/metastatic osteosarcoma between January 2005 and May 2011 were reviewed retrospectively. Of these patients, 21 were administered the gemcitabine plus docetaxel combination, and 18 were provided the pemetrexed plus cisplatin combination. Treatment was continued until the occurrence of disease progression or unacceptable toxicity. In the gemcitabine plus docetaxel group, the overall response rate and disease control rate were found to be 9.5 and 28.5% respectively, compared with 5.5 and 33.3% respectively in the pemetrexed plus cisplatin group. The median progression-free survival (PFS) time was found to be 1.8 months for both the gemcitabine plus docetaxel and pemetrexed plus cisplatin groups. The median overall survival (OS) time was 6 months in the gemcitabine plus docetaxel group and 7 months in the pemetrexed plus cisplatin group. No statistically significant differences were recognized between the overall response rates, disease control rates, PFS times and OS times in the two groups. The two combinations appeared to be well tolerated. However, the incidence of grade 3/4 thrombocytopenia and leucopenia was higher in the gemcitabine plus docetaxel group than in the pemetrexed plus cisplatin group. The present study clearly demonstrated that both chemo-combinations were well-tolerated and exerted antitumor activity in patients with refractory/metastatic osteosarcoma. However, with regard to grade 3/4 toxicity, the pemetrexed plus cisplatin chemotherapy appears to be better tolerated.

## Introduction

Osteosarcoma is the most frequent type of malignant primary bone tumor in childhood (1). The prognosis for localized extremity osteosarcoma treated with surgery alone is poor, with a 2-year survival rate of <20%, as the tumor exhibits a high propensity to metastasize to the lungs (2). Although there have been developments in treatment with the administration of large doses of adjuvant/neoadjuvant chemotherapy, 30% of patients with localized disease and 80% of patients with metastatic disease at diagnosis suffer relapse (3,4). In addition, the failure of standard multimodal therapy in osteosarcoma is common. However, surgical resection has been demonstrated to prolong survival times in osteosarcoma patients, particularly among those with pulmonary metastases (5–8). Furthermore, stereotactic radiosurgery has been effective in certain osteosarcoma patients with pulmonary metastases (9). However, neither the value of second-line chemotherapy nor the best treatment strategy for relapsed patients with high-grade, advanced or metastatic disease has been well-defined (10). This is further complicated by the fact that there are a limited number of studies concerning the management of patients with relapsed osteosarcoma, due to the relative rarity of the disease. Consequently, outcomes such as tumor response rate following second-line treatment are not well-established; therefore, metastatic osteosarcoma remains difficult to treat. Since a lack of consensus has been reached regarding the choice of second-line therapy, the efficacy of novel agents is being continuously evaluated. Although several novel agents, including pirarubicin (11), topotecan (12), irinotecan (13), imatinib mesylate (14) and temozolamide (15) have been investigated, the response rates remain low and survival times short.

Gemcitabine, a difluorinated deoxycytidine analog, is taken up by cells and is converted to the active diphosphate and triphosphate forms by deoxycytidine kinase. The active forms reduce deoxynucleotide reserves and alter DNA chain elongation, thus gemcitabine induces cell death (16). Docetaxel is a semisynthetic taxane analog of paclitaxel. This molecule induces cytotoxicity by stabilizing microtubules, preventing depolymerization, which results in cell cycle arrest and subsequent apoptosis (17). The gemcitabine plus docetaxel combination has been observed to exert additive and synergistic antitumor effects in patients with recurrent osteosarcoma (18–25).

Pemetrexed is a newly developed antifolate drug that targets multiple enzymes involved in DNA synthesis and folate metabolism. When compared with methotrexate, a drug widely used in the treatment of osteosarcoma, pemetrexed is polyglutamated by folylpolyglutamate synthase at 90 to 200 times greater efficiency (26). A phase II trial has previously demonstrated the efficacy of pemetrexed in soft tissue sarcoma (27), and pemetrexed has also been shown to exert broad-spectrum effects in numerous types of solid tumor (28–30). Pemetrexed has a wider range of activity than methotrexate, the corresponding antifolate predecessor, as pemetrexed exerts effects on multiple targets, and has the ability to affect purine and pyrimidine synthesis (31). Therefore, pemetrexed appears to have potential for osteosarcoma treatment. A phase II study has demonstrated that approximately one-third of patients with relapsed osteosarcoma survived for at least 1 year after pemetrexed treatment (32). Furthermore, several studies have demonstrated that in head/neck cancer and malignant pleural mesothelioma, pemetrexed plus cisplatin combination treatment may prolong survival times in certain patients compared with treatment with pemetrexed or cisplatin alone (33–35). Since cisplatin is one of the most active and widely used drugs in the treatment of high-grade osteosarcoma, the pemetrexed plus cisplatin combination may have improved efficacy compared with cisplatin alone. To investigate the efficacy and safety of the pemetrexed plus cisplatin combination in patients with refractory/metastatic osteosarcoma the present retrospective study was conducted and the results obtained were compared with those of patients receiving the gemcitabine plus docetaxel combination. To the best of our knowledge, no study has been conducted regarding the use of the pemetrexed plus cisplatin combination in osteosarcoma patients, nor any comparison conducted between the pemetrexed plus cisplatin combination and the gemcitabine plus docetaxel combination.

## Materials and methods

### Patient eligibility

Between January 2005 and May 2011, patients with refractory/metastatic osteosarcoma who received the gemcitabine plus docetaxel or pemetrexed plus cisplatin combination as second-line chemotherapy at Shanghai Jiaotong University (Shanghai, China) were selected for this retrospective case series study in accordance with the following criteria: i) diagnosis confirmed histologically; ii) resistance to prior treatment consisting of standard high-grade osteosarcoma chemotherapy agents, including doxorubicin, cisplatin, high-dose methotrexate and ifosfamide (completed >3 weeks prior to trial entry); iii) metastatic and unresectable progressive disease (PD); iv) Karnofsky performance status >70 with life expectancy >3 months; and v) adequate renal, hepatic and hemopoietic function. All enrolled patients exhibited radiological evidence of disease progression prior to the initiation of treatment. Clinical characteristics, including age, gender, pathological subtype and performance status were collected for statistical analysis. Ethical approval for the study was provided by the ethics committee of the Affiliated Sixth People’s Hospital (Shanghai, China) and informed consent was obtained from each patient or patient’s guardian. Information regarding toxicity associated with the administration of chemotherapy was recorded according to the National Cancer Institute (NCI) Common Terminology Criteria for Adverse Events (version 3.0).

### Treatment

The gemcitabine plus docetaxel regimen was administered as follows: Patients initially received gemcitabine at a dose of 675 mg/m^2^ intravenously over 90 min on days 1 and 8 of each 21-day course. The patients also received ondansetron prior to initiation of chemotherapy on days 1 and 8. Gemcitabine administered on day 8 was followed by 75 mg/m^2^ docetaxel administered intravenously over 60 min. To minimize the severity and incidence of hypersensitivity and fluid retention associated with docetaxel, dexamethasone treatment was initiated either the day prior to or the same day as when docetaxel was administered, and was continued for 2 days thereafter.

The pemetrexed plus cisplatin combination was administered as follows: 500 mg/m^2^ pemetrexed was intravenously administered over 10 min on day 1 of a 21-day cycle, followed by 100 mg/m^2^ cisplatin administered intravenously. A preparation of 4 mg dexamethasone was taken orally twice daily on the day prior to, the day of and the day after each administration of pemetrexed. Supplementation with 400 μg folic acid, taken orally, was administered daily 1 week prior to the first pemetrexed treatment and was continued until 3 weeks after experimental therapy was discontinued. At 1 week prior to day 1 of cycle 1, 1,000 μg vitamin B12 was intramuscularly injected and this was repeated every 9 weeks until the study was terminated.

If a patient experienced unacceptable toxicity, treatment was postponed for up to 42 days, initiated at day 1 of any cycle to allow recovery from toxicity until the Common Toxicity Criteria (36) grade 3/4 symptoms had resolved. Subsequently, therapy was resumed at 75% of the previous dosage. Any patient requiring >42 days recovery time or >2 reductions due to toxicity was to be withdrawn from the study.

### Efficacy assessment

Tumor response was usually evaluated in between every two chemotherapy cycles by CT/MRI scan according to the Response Evaluation Criteria in Solid Tumors from the NCI. Treatment responses were classified accordingly as complete response (CR), partial response (PR), stable disease (SD) or PD. The endpoints were to evaluate overall response rate (CR + PR), disease control rate (overall response rate + SD), progression-free survival time (PFS) and overall survival time (OS). PFS and OS times were defined as the intervals between the initiation of treatment and progression of the disease or when the patient succumbed to the disease, respectively. Toxicity was recorded for each cycle of chemotherapy according to the NCI Common Toxicity Criteria grading system (36). Toxicity was classified into four levels, with the following associated factors recorded: i) white blood cell count, ii) platelet count, iii) hemoglobin, iv) gastrointestinal toxicities (nausea and vomiting), v) fatigue, vi) impaired liver function and vii) impaired renal function. The Kaplan-Meier method was employed to compare the PFS and OS results.

## Results

### Patient characteristics

A total of 39 patients with metastatic high-grade osteosarcoma, treated between January 2005 and May 2011, were included in the present study. Of these patients, 21 were provided the gemcitabine plus docetaxel combination with the remaining 18 administered the pemetrexed plus cisplatin combination. The characteristics of patients in the two groups are listed in Table I. Factors such as age, gender, initial tumor site, histotype of tumor, method of surgery for initial tumor and stage were well-balanced between the two groups (all P>0.05).

### Response and outcome

In the gemcitabine plus docetaxel group, 21 patients received up to 56 courses of treatment and at least two cycles of chemotherapy (median, two cycles per patient; range, 2–6). No CR was observed thereafter. The overall response rate and disease control rate were 9.5% (2 out of 21) and 28.5% (6 out of 21), respectively. The median PFS time was 1.8 months and the median OS time was 6 months.

The 18 patients in the pemetrexed plus cisplatin group received up to 48 courses of treatment with at least two cycles of chemotherapy (median, two cycles per patient; range, 2–6). No CR was observed thereafter. The overall response rate and disease control rate were 5.5% (1 out of 18) and 33.3% (6 out of 18), respectively. The median PFS time was 1.8 months and the median OS time was 7 months.

**Figure 1:**
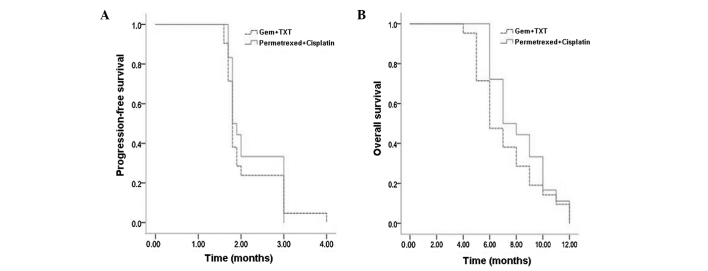
(A) Progression-free survival and (B) overall survival times in the gemcitabine plus docetaxel (Gem+TXT) and pemetrexed plus cisplatin treatment groups.

Subsequent to statistical analysis, no significant differences were identified between overall response rates, disease control rates, median PFS times or median OS times in the two groups (Table II, Fig. 1A and B).

### Toxicity

Table III summarizes the main side effects and all grades of adverse events in the two groups. In general, no drug-related mortality occurred in either group, and chemotherapy-related adverse events were predominantly associated with grade 1 or 2. In 56 courses of gemcitabine plus docetaxel treatment, the observed grade 3 and 4 toxic effects were as follows: 17 cases of leucopenia (30.3%), four cases of anemia (7.1%), nine cases of thrombocytopenia (16.1%), three cases of nausea and vomiting (5.4%) and eight cases of fatigue (14.3%). Conversely, in 48 courses of pemetrexed plus cisplatin therapy, the observed grade 3 and 4 toxic effects were as follows: Two cases of leucopenia (4.2%), one case of anemia (2%), two cases of nausea and vomiting (4.2%) and three cases of fatigue (6.3%). Statistical analysis revealed a significant difference (P<0.05) in the incidence of grade 3/4 thrombocytopenia and leucopenia between the two groups.

Treatment postponement on day 8 occurred in three patients during three of the 56 gemcitabine plus docetaxel combination chemotherapy courses due to a consistent platelet count of <10,000/μl (in one patient) and a consistent WBC count of <1,500/μl (in two patients). No treatment in patients with the pemetrexed plus cisplatin combination had to be postponed due to toxicity.

## Discussion

Patients with refractory/metastatic osteosarcoma have a poor prognosis and novel strategies are therefore required to improve outcomes in this subgroup of patients. In the present retrospective study, treatment of refractory/metastatic osteosarcoma patients with a combination of gemcitabine and docetaxel resulted in a 28.5% disease control rate, a median PFS time of 1.8 months and a median OS time of 6 months. The result was consistent with those of previous studies (24,25). The main adverse reaction of gemcitabine plus docetaxel treatment was grade 3/4 myelosuppression, as detected by leucopenia and thrombocytopenia.

The pemetrexed plus cisplatin combination demonstrated a promising potential in treating refractory/metastatic osteosarcoma. This treatment achieved an efficacy that was parallel to that of the gemcitabine plus docetaxel combination. The adverse reactions following pemetrexed plus cisplatin treatment were well-tolerated with fewer incidences of grade 3/4 toxic events compared with gemcitabine plus docetaxel therapy. Considering that the majority of refractory/metastatic osteosarcoma patients have received large quantities of multi-drug and high-dose chemotherapies (38,39), the potential reserves of bone marrow function in the patients are likely to be poor. Therefore, pemetrexed plus cisplatin treatment may be better tolerated in these patients.

However, the results of the present study are, to some extent, different from those of a recent phase II trial designed to evaluate the efficacy of pemetrexed alone as a second-line chemotherapy in advanced/metastatic osteosarcoma (28). The phase II trial indicated that although it was tolerated well by patients, pemetrexed did not exhibit a good response in refractory/metastatic osteosarcoma (28). This may be the result of certain shortcomings in the present study, such as the retrospective nature and possible patient selection bias, which may affect the prognosis. Such differences may have been due to the supra-additive effect of the pemetrexed plus cisplatin combination. Previous studies have reported that compared with pemetrexed plus cisplatin alone, pemetrexed plus cisplatin treatment achieves an improved prognosis in certain types of cancer (33–35). This ‘synergistic effect’ may be associated with the cisplatin-reinforcement effect of pemetrexed; the patients who benefited from the pemetrexed plus cisplatin combination in the present study had shown drug-resistance during prior treatment, including resistance to cisplatin. Cisplatin enhancement of pemetrexed efficacy is also a possibility. Further studies using the basic model are required to elucidate the underlying molecular mechanism of this supra-additive effect.

The present study had certain shortcomings, namely the retrospective nature, the relatively small number of patients and possible patient selection bias. Despite these limitations, this remains, to the best of our knowledge, the first study describing the effect and toxicity of pemetrexed plus cisplatin in refractory/metastatic osteosarcoma patients, and comparing this treatment regimen with gemcitabine plus docetaxel therapy. Although the two drug combinations were well-tolerated and easily administered, the pemetrexed plus cisplatin treatment appears to have fewer incidences of severe toxicity. To determine whether treatment with pemetrexed plus cisplatin results in longer survival times than those that achieved with pemetrexed alone, prospective studies are required.

## Figures and Tables

**Table I:** Patient characteristics in the gemcitabine/docetaxel and pemetrexed/cisplatin treatment groups.

Clinicopathological parameter	Gemcitabine/docetaxel	Pemetrexed/cisplatin	P-value
Gender			>0.05
Male	15	14	
Female	6	4	
Age (years)			>0.05
<18	11	11	
≥18	10	7	
Location			>0.05
Limbs	18	16	
Nonextremities	3	2	
Histotype			>0.05
Conventional	18	17	
Others	3	1	
Surgical			>0.05
Amputation	7	6	
Limb salvage	14	12	
Metastasis at diagnosis			>0.05
Yes	11	8	
No	10	10	

**Table II:** Tumor responses in the gemcitabine/docetaxel and pemetrexed/cisplatin groups.

Group	No. patients	CR	PR	SD	PD
Gemcitabine/docetaxel	21	0	2	4	15
Pemetrexed/cisplatin	18	0	1	5	12

Patients were classified according to the Response Evaluation Criteria in Solid Tumors: CR, complete response; PR, partial response; SD, stable disease; PD, progressive disease.

**Table III:** Hematological and non-hematological toxicity in patients treated with gemcitabine/docetaxel or pemetrexed/cisplatin.

	Gemcitabine/docetaxel group (56 courses), n (%)			Pemetrexed/cisplatin group (48 courses), n (%)		
		
Toxic event	Grade 1/2	Grade 3	Grade 4	Grade 1/2	Grade 3	Grade 4
Anemia	32 (57.1)	3 (5.3)	1 (1.8)	21 (43.7)	1 (2.0)	0 (0.0)
Leucopenia	30 (53.5)	12 (21.4)	5 (8.9)	11 (22.9)	2 (4.1)	0 (0.0)
Thrombocytopenia	26 (46.4)	7 (12.5)	2 (3.6)	8 (16.7)	0 (0.0)	0 (0.0)
Nausea and vomiting	18 (32.1)	3 (5.3)	0 (0.0)	20 (41.7)	2 (4.1)	0 (0.0)
Fatigue	22 (39.2)	6 (10.7)	2 (3.6)	21 (43.7)	3 (6.3)	0 (0.0)
Impaired liver function	5 (8.9)	0 (0.0)	0 (0.0)	12 (25.0)	0 (0.0)	0 (0.0)
Impaired kidney function	2 (3.5)	0 (0.0)	0 (0.0)	1 (2.0)	0 (0.0)	0 (0.0)
Alopecia	12 (21.4)	0 (0.0)	0 (0.0)	13 (27)	0 (0.0)	0 (0.0)

Toxicity was graded according to the National Cancer Institute Common Terminology Criteria for Adverse Events (version 3.0) (37).
